# The Treatment of Infectious Diseases Arising in Children's Hospitals

**Published:** 1896-12-26

**Authors:** Alfred Parkin

**Affiliations:** Senior Surgeon to the Victoria Hospital, Hull.


### THE TREATMENT OF INFECTIOUS DISEASES ARISING IN CHILDREN'S HOSPITALS.

By ALFRED PARKIN, M.S., M.D.(Lond.), Senior Surgeon to the Victoria Hospital, Hull.

Probably nothing is more annoying to a visiting surgeon than to find that one or more of the cases under his care has developed symptoms of an infectious disease, and, unfortunately, such an occurrence is only too common in children's hospitals. The susceptibility of children to such diseases on the one hand, and the obvious fact that a patient can be admitted for a trifling affection whilst actually incubating an infectious disease, on the other hand, are the chief reasons for this. No matter how much care be taken in the selection of cases, it is not infrequently found that children admitted with a high temperature, without other symptoms, have subsequently to be removed or isolated. If to these we add the risk of infection through contact with parents, brothers, and sisters, it becomes almost a matter for wonder how it is that outbreaks are not more frequent.

It is practically impossible to ask all visitors if the neighbourhood from which they come is at all infected, and it is to be feared that the natural anxiety of a parent to see its child often prevents a true answer being given when such a question is asked. The entire abolition of visiting-day would be an excellent step, only this would not be tolerated except under the exceptional circumstances of an epidemic; but the less severe step of preventing children from accompanying the adult visitors would in all probability be productive of much good.

The difficulties of diagnosis are often very great, especially in very young children. Sweat rashes and fugitive erythemata are common, with or without rises of temperature, and frequently the diagnosis follows the wish rather than the thought. The diagnosis of measles before the appearance of the rash is a matter of difficulty, especially if unsuspected, and it is more particularly during the period of uncertain diagnosis that the surrounding patients are liable to become infected. It is fortunate that measles has a comparatively short duration, otherwise our difficulties would be very much increased.

When many cases have broken out in one ward the diagnosis often becomes more difficult from abnormal or atypical cases. As an instance of this I might mention a girl under my care for morbus coxæ, who developed hæmaturia seven or eight weeks after an outbreak of scarlatina had arisen, which we all thought had been quickly suppressed. This patient two days later began to have convulsions, and died in about thirty hours. On referring to her temperature chart it was seen that she had had on one occasion a rise of temperature to 101°, five weeks before, but there had been no other symptoms whatsoever. Post-mortem, the kidneys were affected with acute nephritis.

It is unfortunate that measles is considered by most people to be an almost necessary disease of childhood, of slight importance only. As a consequence its treatment is neglected amongst the poorer classes, notification is seldom rendered compulsory, reception in a sanitorium is not provided for, and, as a result, it rapidly becomes epidemic, and the death-rate rises from the secondary affections—broncho-pneumonia, whooping-cough, otitis media, &c.

Whilst it is the obvious duty of the medical officers of an institution to prevent the introduction of infectious disease amongst the inmates, it is no less the duty of the hospital authorities to provide all necessary means for preventing the spread of the disease, and for the proper isolation of the affected inmates when an outbreak unfortunately occurs. It would be very advantageous if every children's hospital had a small but distinct block, one room of which could be got ready at an hour's notice to receive any doubtful case, rather than allowing such a case to develop in a general ward. A similar room might be used with advantage for the admission of cases of diphtheria requiring surgical treatment, and the rest of the building could be adapted for the reception of the patients from a general ward, when, owing to a number of cases having developed in it, thorough measures of disinfection and cleansing have to be resorted to.

In this way it would be easy to avoid closure of a ward in the hospital itself, or, what is even worse, closure of the whole hospital. Such a course is, in reality, a most expensive one, for the working expenses of a hospital are almost the same whether three or four wards are kept open, and the return for money spent is very much reduced.

In towns provided with a sanitorium, or hospital for infectious diseases, it is easy to provide for the removal of the majority of cases arising in a general hospital; but even then there are cases that have developed infectious diseases subsequent to operations, and a small proportion of medical cases which are not fit to be removed. These must of necessity remain in the hospital until they can be safely removed, and consequently the provision of an isolated room or ward is a necessity.

To permit a patient to remain in a general ward, when such a patient is infectious to others, or even liable to become so, ought under no circumstances to be allowed, no matter what precautions are taken; isolation under these circumstances by carbolised screens is of little use, as the patient has of necessity to be attended to by nurses and the house surgeon, who may immediately attend to other patients, with or without the adoption of any precautionary measure, even without washing their hands.

It will, I think, be evident to everyone that the expense of immediate and complete isolation of an infectious patient is really nothing compared with the expense likely to be incurred by such infection spreading through a ward, or becoming general in a hospital; and if to the expense be added the risk to life, and the danger of complications immediate and remote, to all the inmates and their relations, we have a powerful series of arguments for the prompt and effective isolation of all such cases, as they occur, in a properly-constructed building or block.

When no infectious diseases hospital exists in the neighbourhood, or where the town provides no accommodation for measles, the only means that can be taken in such cases is to promptly remove them, as they occur, to the isolated portion of the hospital. The only alternative is to send the patients back to their own homes. Such a proceeding, especially when the infection was caught in the hospital itself, can only be regarded as iniquitous, most unfair to the patient and the patient's friends, and liable to spread infection in a wholesale manner.

In most towns the mortality from whooping-cough and its results is considerable, and yet practically no provision is made for the treatment of the very poor, whose children are so often victims to this disease. Such children frequently come up to the out-patient department of a children's hospital, and so come in contact with surrounding patients. If a case originates inside the hospital, it is promptly sent home, with the inevitable result of conveying infection to others in the neighbourhood.

From a consideration of the above points it is evident that a properly-constructed children's hospital ought to be provided with adequate means for the immediate isolation of any infectious cases, and, if possible, with a separate department for the treatment of whooping-cough; this is necessary, if only on the ground of saving expense.

The responsibility of founding and trying to work a hospital without such a provision is so serious a matter that it would never be undertaken, were the whole question understood or investigated in a business-like spirit.

